# A systematic review of studies on forecasting the dynamics of influenza outbreaks

**DOI:** 10.1111/irv.12226

**Published:** 2013-12-23

**Authors:** Elaine O Nsoesie, John S Brownstein, Naren Ramakrishnan, Madhav V Marathe

**Affiliations:** a Children's Hospital Informatics Program, Boston Children's HospitalBoston, MA, USA; b Department of Pediatrics, Harvard Medical SchoolBoston, MA, USA; c Network Dynamics and Simulation Science Laboratory, Virginia Bioinformatics Institute, Virginia TechBlacksburg, VA, USA; d Department of Epidemiology, Biostatistics and Occupational Health, McGill UniversityMontreal, QC, Canada; e Department of Computer Science, Virginia TechBlacksburg, VA, USA

**Keywords:** Compartmental models, individual-based models, infectious diseases, influenza forecasting, pandemics, time series models

## Abstract

Forecasting the dynamics of influenza outbreaks could be useful for decision-making regarding the allocation of public health resources. Reliable forecasts could also aid in the selection and implementation of interventions to reduce morbidity and mortality due to influenza illness. This paper reviews methods for influenza forecasting proposed during previous influenza outbreaks and those evaluated in hindsight. We discuss the various approaches, in addition to the variability in measures of accuracy and precision of predicted measures. PubMed and Google Scholar searches for articles on influenza forecasting retrieved sixteen studies that matched the study criteria. We focused on studies that aimed at forecasting influenza outbreaks at the local, regional, national, or global level. The selected studies spanned a wide range of regions including USA, Sweden, Hong Kong, Japan, Singapore, United Kingdom, Canada, France, and Cuba. The methods were also applied to forecast a single measure or multiple measures. Typical measures predicted included peak timing, peak height, daily/weekly case counts, and outbreak magnitude. Due to differences in measures used to assess accuracy, a single estimate of predictive error for each of the measures was difficult to obtain. However, collectively, the results suggest that these diverse approaches to influenza forecasting are capable of capturing specific outbreak measures with some degree of accuracy given reliable data and correct disease assumptions. Nonetheless, several of these approaches need to be evaluated and their performance quantified in real-time predictions.

## Introduction

An extensive body of the literature exists on mathematical and computational models for studying the spatio-temporal dynamics of influenza outbreaks. A main purpose of some of these models is to inform public policy regarding the selection and allocation of public health interventions and resources during a pandemic.^1^ Reliable forecasts of measures such as peak time, peak height, and magnitude during an outbreak would inform public health practitioners and healthcare workers on when to expect a surge in demand for healthcare resources and infrastructure and the overall expected public health impact of an outbreak. Although timely forecasts of these measures would be beneficial, making reliable predictions during an outbreak remains a public health challenge.

Several of the major approaches applied to modeling influenza transmission and dynamics have been applied to the forecasting of influenza outbreaks (see Table 1 for brief descriptions).^2^–^5^ These models have been reviewed in the context of pandemic preparedness, control, and mitigation.^1^,^6^–^8^ However, there are no reviews discussing the application of these models to the forecasting of influenza outbreaks. The goal of this paper is therefore to present a systematic review of studies that have discussed approaches for influenza forecasting at the local, regional, national, or global level. The main aims are to (i) summarize existing approaches to influenza forecasting, (ii) present differences in measures of accuracy and evaluate the degree to which various performance measures are met, (iii) discuss limitations in the data sources, and parameter estimation that impede forecasting during outbreaks. The motivation of this paper is to inform further research on influenza forecasting and provide researchers and public health practitioners with a summary of the accomplishments and limitations in influenza forecasting.

**Table 1:** Model description, advantages, and limitations

Approach	Description	Advantages	Limitations
Time series models	The Box-Jenkins approach, specifically the autoregressive integrated moving average (ARIMA) model is typically used. ARIMA models assume that future values can be predicted based on past observations.	ARIMA models capture lagged relationships that usually exist in periodically collected data. In addition, temporal dependence can also be adequately represented in models that are capable of capturing trend and periodic changes.	Influenza activity is not consistent from season to season, which could impose limitations to ARIMA models, especially during pandemics, which can occur off-season.
Approaches in meteorology (Method of analogs)	The method of analogs is a nonparametric forecasting approach in meteorology. Forecasting is based on matching current influenza patterns to patterns of historical outbreaks.	The onset of seasonal influenza epidemics varies from year to year in most countries in the Northern hemisphere. As the method of analogs is nonparametric, implying it makes no assumptions on underlying distributions or seasonality, it can sometimes outperform methods (such as ARIMA) that include a seasonal component.	Limitations exist on the sensitivity to forecasts and difficulty in finding similar patterns from historical outbreaks.
Compartmental models	These models divide the population into compartments based on disease states and define rates at which individuals move between compartments. Examples include susceptible–infectious–recovered (SIR) and susceptible–exposed–infectious–recovered (SEIR) models.	Compartmental models are attractive due to their simplicity and well-studied behavior. These models are typically extended by defining multiple compartments to introduce subpopulations, including a branching process, or used in combination with other approaches, such as particle filtering, for influenza forecasting.	The usual fully mixed, homogenous population assumption fails to capture the differences in contact patterns for different age groups and environments.
Agent-based models	These are computational systems in which the global behavior emerges due to individual behavior of well-defined entities called agents, which interact with other entities and their environment based on specific rules.	These models have been used to address questions relating to the impact of control measures and changes in individual behavior during an outbreak. They can therefore enable the forecasting of influenza dynamics under different intervention and resource allocation scenarios.	One major difficulty in applying these models is the rather circumscribed assumptions under which they operate, compounded by our limitations in understanding the modeling of human behavior via contact networks.
Metapopulation models	Populations in the model are represented in structured and separated discrete patches and subpopulations interact through migration. Epidemic dynamics can be described within patches using clearly defined disease states such as in compartmental models.	The detailed mobility networks used in some of these models can enable reliable description of the diffusion pattern of an ongoing epidemic. These models have also been used to evaluate the effectiveness of various measures for controlling influenza epidemics.	Similar to agent-based models, there exist the challenge of empirically justifying modeling suppositions and defining parameters.

## Article selection and evaluation

The scope of this review included studies designed to predict influenza dynamics at the local, regional, national, or global level. First, we searched PubMed and Google Scholar for articles on influenza forecasting. A search for (“influenza, human”[MeSH Terms] OR (“influenza”[All Fields] AND “human”[All Fields]) OR “human influenza”[All Fields] OR “influenza”[All Fields]) AND (“forecasting”[All Fields] OR “forecasting”[MeSH Terms]) on PubMed retrieved 239 articles. Replacing “forecasting” with “prediction” in the previous search criteria resulted in 370 articles. A Google Scholar search for “influenza forecasting” retrieved 12 000 articles. Next, we focused on articles with “influenza” and “forecasting” or “prediction” in the titles and/or abstracts. Third, we selected articles that mentioned influenza forecasting as one of the aims in the abstract. After eliminating non-English articles, 35 articles remained. Lastly, we excluded articles focusing on topics such as forecasting emergency department visits, which have already been covered in a previous review.^33^ The study is therefore based on the remaining 16 articles, which included both prospective and retrospective studies. We group and present studies based on measures predicted.

## Results

We acknowledge that there were numerous endeavors made by various research groups and organizations toward real-time forecasting of the 2009 H1N1 pandemic. However, for several of these endeavors, we were unable to find published descriptions of the methodology used in forecasting. A brief description of the modeling approaches in the sixteen selected articles, in addition to advantages and limitations to using these methods for influenza forecasting can be found in Table 1. In Table 2, we present a summary of study characteristics.

**Table 2:** Summary of study characteristics

Author	Publication year	Date type	Data scale	Data range	Location	Method	Predicted measure	Measure of accuracy
Longini *et al*.^15^	1986	ILI	Weekly	1968–1969	52 cities	Mathematical model defined on a continuous state space in discrete time	ILI across 425 days and peak period	Deviation from ILI estimated based on WHO reports
Aguirre & Gonzalez^9^	1992	ILI	Daily	1988	Havana, Cuba	Mathematical model defined on a continuous state space in discrete time	Daily ILI, peak, and duration	Correlation and statistical tests
Viboud *et al*.^10^	2003	ILI	Weekly	1984–2002	France & Administrative Districts	Method of analogs	Weekly ILI	Correlation and RMSE
Hall *et al*.^3^	2006	ILI and deaths attributable to influenza	Weekly	1968–1970, 1918–1919 & 1957–1958	United Kingdom	Deterministic mass action model	Timing and amplitude of peak, duration, and magnitude	Error and time difference
Polgreen *et al*.^13^	2007	Influenza activity	Weekly	2004–2005	Iowa, USA	Prediction markets	Weekly activity based on CDC's color coded system	Proportion predicting correct color code
Andersson *et al*.^20^	2008	LCI cases	Weekly	1999–2006	Sweden	Regression model and prediction rules	Peak timing and height	Error and time difference
Jiang *et al*.^11^	2009	ILI and deaths attributable to influenza	Daily	2006	USA	Bayesian network	Epidemic curve	Correlation and error
Towers & Feng^16^	2009	Influenza case count data	Weekly	2009	USA	SIR model	Peak time and attack rate	Confidence intervals
Soebiyanto *et al*.^12^	2010	LCI cases	Weekly	2005–2008	Hong Kong & Maricopa county, AZ, USA	ARIMA model	Weekly case counts	RMSE
Ong *et al*.^4^	2010	ILI	Weekly	2009	Singapore	SEIR model with particle filtering	Weekly case counts, peak timing, and duration	Error
Chao *et al*.^2^	2010	CDC influenza case estimates and estimates of vaccine availability and distribution	None	2009–2010	USA & LA County, USA	Epidemic simulation model based on a synthetic population	Peak timing and magnitude	Predicted range
Nishiura^14^	2011	Influenza cases	Weekly	2009–2010	Japan	Discrete time stochastic model	Weekly case counts	Prediction intervals
Shaman & Karspeck^18^	2012	Google Flu Trends	Weekly	2003–2008	New York City, USA	SIRS model with ensemble adjustment Kalman filter	Peak timing	Posterior estimates and deviation
Tizzoni *et al*.^5^	2012	ILI, ARI incidence, LCI	Weekly	2009	48 countries	Metapopulation stochastic epidemic model	Peak timing and attack rate	Confidence intervals and time difference
Hyder *et al*.^21^	2013	LCI	Weekly	1998–2006	Montreal, QC, Canada	Individual-based model	Peak timing, peak intensity, and epidemic duration	Error and time difference
Nsoesie *et al*.^19^	2013	Google Flu Trends	Weekly	2004–2005, 2007–2008 & 2012–2013	Seattle, WA, USA	Individual-based model	Peak timing	Confidence intervals and deviation

LCI, laboratory confirmed influenza; ILI, influenza-like illness; ARI, acute respiratory infection; ARIMA, autoregressive integrated moving average; RMSE, root-mean-squared-error.

### Measures predicted

The articles in Table 2 aimed to either forecast a single measure or multiple measures. Typical measures predicted included epidemic trend, duration, peak timing, peak height, and magnitude. For simplicity, we grouped these measures into magnitude, peak timing and intensity, and duration. We discuss differences in measures of accuracy, which appeared to depend on the modeling approach and the measure predicted.

#### Magnitude

Eleven of the sixteen studies forecasted the expected magnitude, daily or weekly influenza activity based on data on confirmed laboratory cases, and/or influenza-like illness. As noted, measures of accuracy differed across studies. Aguirre and Gonzalez,^9^ Viboud *et al*.^10^, and Jiang *et al*.^11^ used correlation coefficients to evaluate accuracy in daily and weekly forecasts of influenza activity. The correlation coefficient between the predicted and observed values ranged from 58% to 93·5% depending on the length of the forecasts. Although useful in comparing data trends, correlation coefficients do not measure the closeness of the predicted to the observed values.

On the other hand, the closeness of the predicted to the observed data could be evaluated using different measures of error. For instance, Jiang *et al*.^11^ observed different percent errors depending on when prediction was made. Prediction of the epidemic curve made a few days from the peak had an estimated 10·8% percent error, which was much lower than the 91·6% percent error observed using nine fewer data points. Similarly, Soebiyanto *et al*.^12^ presented several ARIMA models and evaluated accuracy based on the root-mean-squared-error (RMSE) of one-step-ahead predictions. They also considered the effects of including environmental variables such as humidity and temperature. The preferred models had RMSE approximately in the range of 0·47–0·61. Alternatively, Polgreen *et al*.^13^ presented a prediction market for influenza forecasting and assessed accuracy based on the proportion of correct predictions of a particular color code representing a level of influenza activity. The prediction markets yielded correct predictions 71%, 50%, and 43% of the time by the end of the target week, 1 week in advance, and 2 weeks in advance, respectively.

Some of the studies evaluated accuracy using prediction and confidence intervals. For instance, the true incidences were included in the 95% prediction intervals for epidemic forecasts made at the peak and after the peak for the 2009 pandemic in Japan by Nishiura.^14^ Predictions made for the 1968–1969 pandemic, also known as the Hong Kong flu, were presented graphically and assessed to have overlapped with the observed data in 42 of 44 cities.^15^ Influenza case estimates made by Chao *et al*.^2^ also overlapped with the estimated ranges from the US CDC.

Most of the previous methods were evaluated retrospectively or published after the 2009 pandemic. Towers and Feng^16^ presented forecasts of the 2009 pandemic in the US as it unfolded. They predicted the proportion of the infected population at 63% without vaccination and 57% with the inclusion of the planned vaccination scheme in the model. The 57% estimate was much higher than estimates presented by the CDC. However, real-time predictions of outbreak dynamics are extremely difficult compared with retrospective evaluations due to limitations in data and difficulty in obtaining reliable parameter estimates as we later discuss.

#### Peak timing and intensity

Methods applied to forecasting peak time have been shown to perform reasonably well when reliable data and parameter estimates are used. For instance, during the 2009 pandemic, Towers and Feng^16^ predicted that the peak would be observed in the US toward the end of October in week 42 with 95% confidence intervals between weeks 39 and 43. According to CDC reports,^17^ H1N1 peaked in the US during the second week of October. Ong *et al*.^4^ also predicted a few weeks in advance that the 2009 pandemic in Singapore would peak at the start of August. However, the peak height was overestimated. Chao *et al*.^2^ also showed that simulated 2009 H1N1 epidemic for LA County peaked at about the same time (mid-November) as reported by the LA county Department of Public Health.

Using web-based estimates of influenza activity, Shaman and Karspeck^18^ and Nsoesie *et al*.^19^ retrospectively illustrated that peak time could be predicted as early as 7 and 6 weeks, respectively, before the actual peak for seasonal outbreaks of influenza in the US. Unfortunately, web-based estimates do not always capture trends in influenza activity and could therefore distort accuracy of predicted outcomes.

Studies published before the 2009 pandemic also had some success. For example, the model discussed by Longini *et al*.^15^ retrospectively estimated the peak time for the 1968–1969 Hong Kong influenza pandemic within the 4-day epidemic peak period for 32% of the cities for which morbidity data were available. Using the same model as that discussed in, Longini *et al*.,^15^ Aguirre and Gonzalez^9^ predicted the 1988 influenza epidemic in Havana, Cuba to peak on March 15th. However, the true peak was observed on March 1st, implying a deviation of approximately 2 weeks. Additionally, Hall *et al*.^3^ showed that pandemic amplitude could be predicted to within 20% and peak timing within a week in retrospective evaluations using ILI and mortality data for three pre-2009 pandemics. Andersson *et al*.^20^ observed a median error of 0·9 weeks and a median deviation of approximately 28% for predictions of the peak time and peak height, respectively, for seven seasonal outbreaks (from 1999 to 2006) in Sweden.

Compared with the other metrics, the peak time appears to be the easiest to forecast. However, forecasting the peak height is more complex and is usually over- or underestimated.

#### Duration

Outbreak duration is typically defined in terms of baseline levels of infection. Compared with the other metrics, fewer papers have focused on predicting outbreak duration. Aguirre and Gonzalez^9^ correctly predicted the end of the 1988 epidemic in Havana, Cuba. Based on a retrospective study of three pandemic events, Hall *et al*.^3^ predicted pandemic durations within 2 weeks of the actual duration. In contrast, Hyder *et al*.^21^ retrospectively illustrated that duration could be underestimated by as little as 2 weeks and as much as 14 weeks for some influenza seasons.

The previously discussed results suggest that reliable forecast of influenza dynamics is possible. However, diversity in modeling approaches, and differences in measures of accuracy makes forecast comparison difficult.

## Discussion

The number of new infections at any time during an influenza outbreak depends on several biological, behavioral, and environmental factors that influence the transmission of influenza viruses.^22^ These factors include immunity, virulence factors, contact type and patterns, and climatic conditions that influence viral survival. The inclusion of these parameters in models for influenza forecasting could improve forecast accuracy. However, in addition to the difficulty of estimating true influenza incidence from laboratory confirmed cases and ILI, estimating transmission and severity parameters during pandemics remains a challenge.^23^ We discuss these challenges.

### Parameter estimation

Unlike seasonal outbreaks of influenza, pandemics are rare and usually result from novel influenza viruses. A meager understanding of the natural history of the virus hinders the estimation of transmission and severity parameters in real time. Estimating the transmission potential of an emerging infection early on is important as it would help determine whether control measures should be varied and whether more stringent measures are required to control or mitigate an outbreak.^24^,^25^ In several publications, the transmissibility and natural history of influenza have been estimated at the household, school, or community level using observational data.^26^,^27^ However, data are typically unavailable or incomplete during the early stages of an outbreak resulting from a novel influenza virus.

The disease severity, which is another important measure, is commonly estimated based on case fatality, hospitalization rates, and clinical attack rates. Approximations of case fatality and hospitalization rates could be underestimated due to subclinical and asymptomatic cases. Although clinical attack rates could be estimated at the community level, data on laboratory-diagnosed cases might be delayed. Nevertheless, studies conducted during the 2009 pandemic suggested that estimates of severity and transmissibility improved as the pandemic progressed.^27^,^28^

### Data

Traditional systems for monitoring ILI and acute respiratory tract infections rely on reports from general practices, family doctor clinics, diagnostic test laboratories, and public health departments for influenza surveillance.^3^,^4^,^14^ There is typically 1–2 week lag(s) in the publishing of reports, and reported cases are sometimes retrospectively adjusted. Additionally, the exact number of influenza cases is unobtainable due to unreported cases and asymptomatic infections.

In view of the challenge in obtaining timely influenza surveillance data from conventional methods, alternative sources of data such as Google Flu Trends have been considered. Google Flu Trends^29^ attempts to provide estimates of influenza activity based on Internet search data. Other data sources, such as flu prescription drug sales, nonprescription medication sales, school absenteeism, ILI symptom reports on social media, and emergency department chief complaints, have also been evaluated as proxies for capturing ongoing influenza trends.

Although these novel data sources provide information in near real time, which is useful for daily or weekly forecasts of influenza activity,^18^,^19^,^30^ there are several limitations to using these data. Limitations include reduced application in low-resource countries and deviations from influenza patterns presented by traditional surveillance systems. For example, Cook *et al*.^31^ compared H1N1-related search queries on Google Insight to traditional surveillance data for the H1N1 pandemic in Singapore. The outbreak peaked in August 2009; however, search query data suggested an earlier peak and also decreased to about 20% of the search volume around the epidemic's peak time. Furthermore, during the 2012–2013 influenza season, estimates of influenza activity provided by Google Flu Trends did not match estimates provided by traditional influenza surveillance systems.^32^ The challenge therefore remains for timely estimates of influenza activity for weekly forecasts at different geographical levels.

## Conclusion

**Figure 1:**
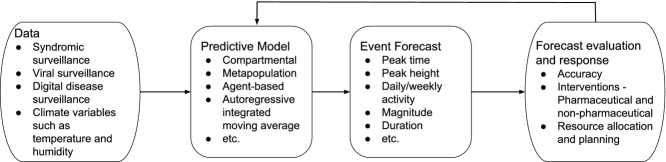
Summary of forecasting process.

Reliable forecasts of measures such as trend, peak height, and peak time during influenza outbreaks would inform healthcare practitioners on when to expect changes in demand for healthcare resources. Practitioners could therefore prepare for surges in influenza cases by acquiring the necessary resources (such as vaccines and antiviral treatments) and alerting essential personnel (such as nurses and doctors). However, forecasts must be interpretable to be useful. It is therefore important for studies to clearly define the predicted event, the temporal and spatial applicability of the approach, quantify the likelihood of the event either based on a probabilistic statement or relative to other similar events, and highlight the limitations (see Figure 1). In addition, defining a global measure of accuracy for evaluating the correctness of various forecasting methods would ease the process of forecast comparison. Lastly, several of the studies discussed in this review are retrospective. The challenge therefore remains in evaluating and quantifying the performance of these methods in real time.
